# Acute Avulsion of the Iliac Crest Apophysis in an Adolescent Indoor Soccer

**DOI:** 10.5334/jbr-btr.876

**Published:** 2015-12-30

**Authors:** Bruno Coulier

**Affiliations:** 1 Clinique Saint-Luc, Bouge, Belgium

**Keywords:** Apophyseal fracture, avulsion fracture, apophysis, pelvis, iliac crest

## Abstract

We report a typical case of acute avulsion of the anterior iliac crest apophysis diagnosed in an indoor football player. The injury occurred as a result of a sudden twist of the trunk while kicking. Plain radiographs made the diagnosis. Complementary CT with 3D reconstructions was preferred to ultrasound because of the very strong habitus – 110 kilograms for 1,73 meter – of the 15-year old adolescent. CT confirmed that occult chronic mechanical stress on the iliac apophysis had preceded the acute avulsion and also emphasized the crucial role of the tensor fascia lata in the mechanism of the injury. The patient was successfully treated conservatively. The case is presented with a short review of the literature.

## Case report

A 15-year old Turkish adolescent was admitted in the emergency department one hour after injuring his left hip while playing indoor soccer. The patient was neither able to walk nor bear weight. The area of the left iliac crest was painful and active mobilization of the left hip was totally impossible. This young right-handed patient had a very strong habitus, weighing 110 kilograms for 1, 73 meter. He explained that he had kicked the ball fast with his dominant right leg at a sharp angle to the left so that he had to rotate his pelvis far to the left while maintaining the upper trunk to the right. At the end of this movement the patient suddenly felt an acute painful crack at the left iliac crest followed by a fall and a total inability to stand upright.

**Figure 1:**
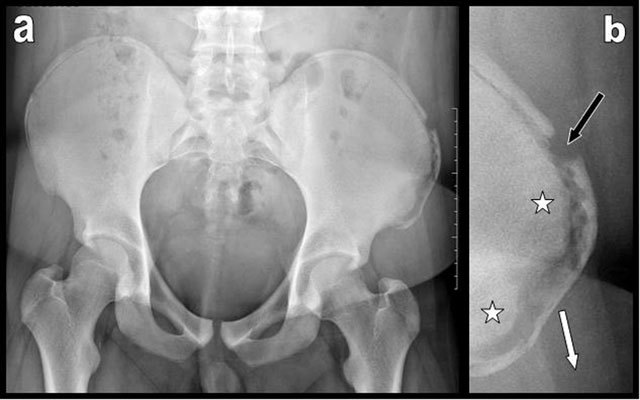
Pelvic plain film (a) and focused view (b) of the left iliac crest illustrate avulsion of the anterior part of the left iliac crest apophysis. Diastasis between the parent bone and the apophysis is clearly visible. The apophysis is displaced outwardly and downwardly (white arrow). Transverse fracture of the iliac crest apophysis itself is associated (black arrow). Preexisting bony sclerosis of the parent iliac bone due to chronic overuse is visible (white stars).

**Figure 2:**
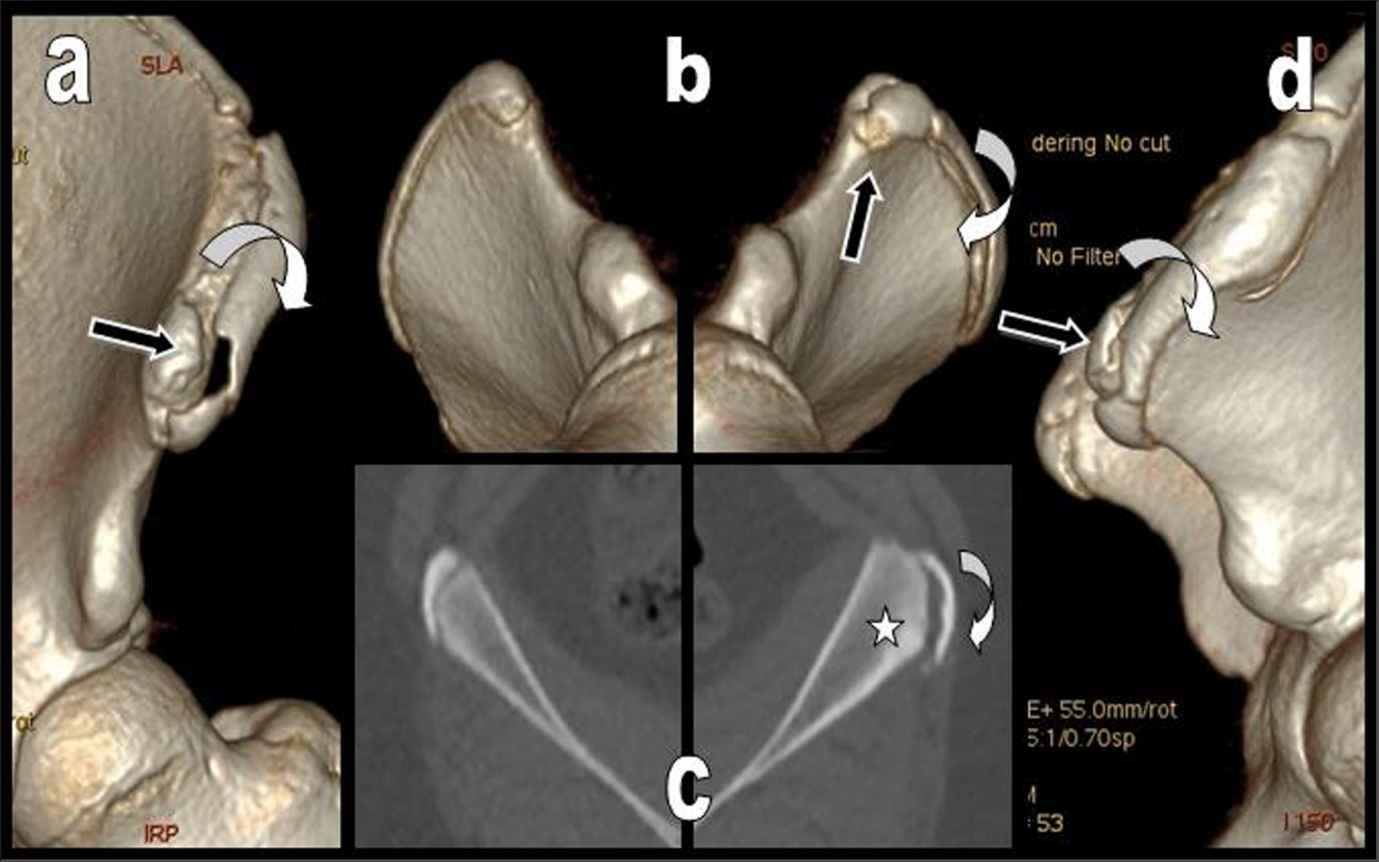
Left anterior (a), comparative right and left oblique inferior (b) and left lateral (d) 3D CT reconstructions of the left iliac crests illustrate the avulsion of the anterior part of the left iliac crest apophysis. It is displaced outwardly and downwardly (curved white arrow) but the apophysis of the anterior and superior iliac spine - origin of the sartorius - is respected and not displaced (black arrow). Comparative axial views of the right and left iliac crests (c) show preexisting hypertrophy and bony sclerosis of the left parent iliac bone due to chronic overuse (white star).

**Figure 3:**
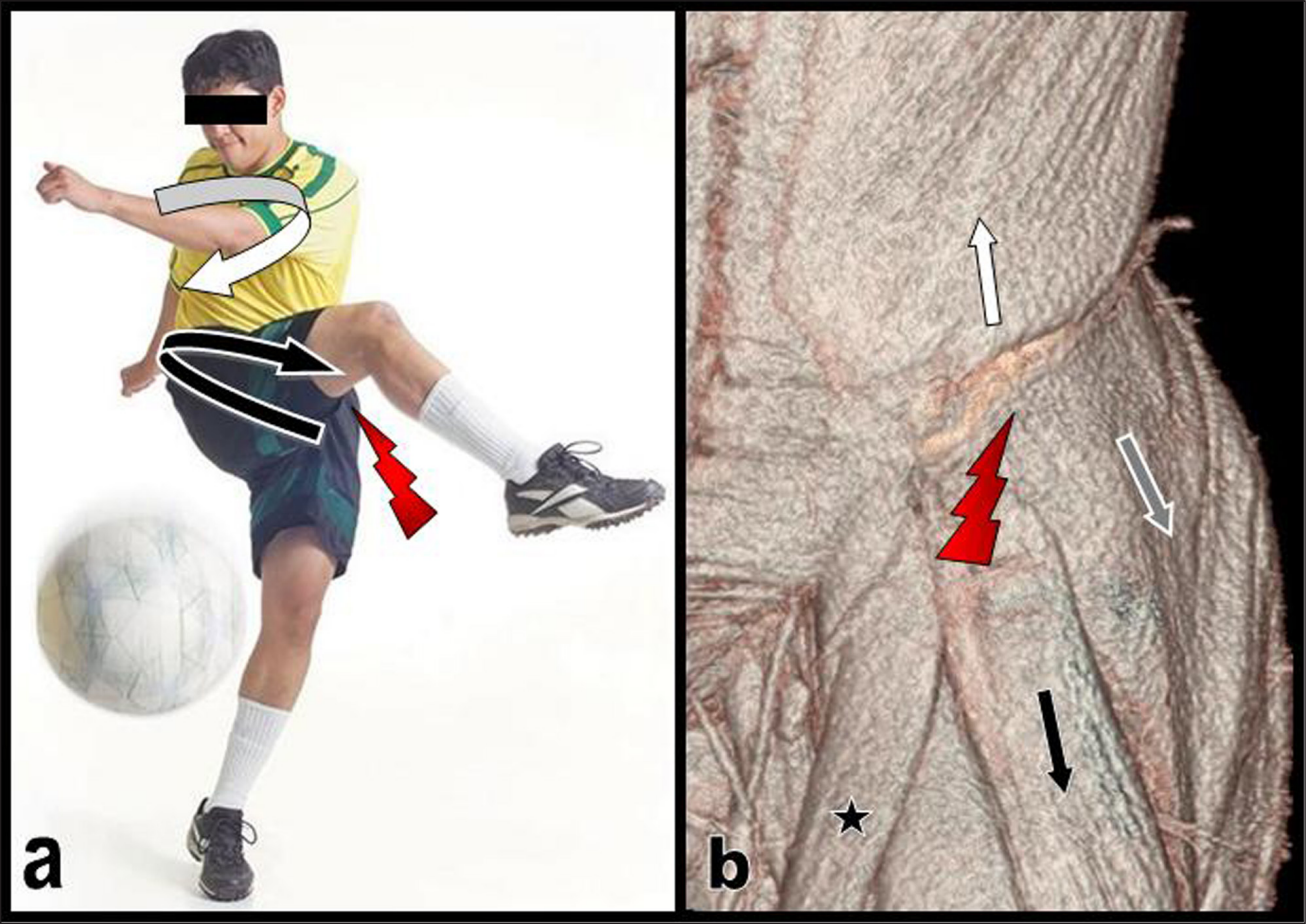
Typical posture of a right-handed soccer during fast kicking (a) volume rendering CT view of the muscles inserting on the iliac crest of our patient (b). Kicking is initiated by rotating the pelvis far to the left (curved black arrow on a) around the supporting left leg and by bringing forwards the thigh of the right kicking leg while maintaining the upper trunk to the right (curved white arrow on a). The sudden traction and contraction of the left external oblique (white arrow) probably pulls the apophysis off the left iliac crest. The action of the external oblique muscle is abruptly thwarted (red flash) by the antagonist traction of the gluteus medius (grey arrow) but merely of the tensor fascia lata (black arrow) that displaces the avulsed apophysis outwardly and downwardly. Black star = Sartorius.

Plain film of the pelvis demonstrated the avulsion of the anterior segment of the left iliac crest apophysis (Figure 1). Complementary CT with 3D reconstructions (Figure 2) was preferred to ultrasound because of the very strong habitus of the patient. Avulsion of the iliac crest apophysis with respect of the anterior superior iliac spine apophysis was confirmed. Signs of preexisting chronic mechanical bone stress of the parent bone were also found on plain films (Figure 1) but also on axial CT views (Figure 2c). Finally 3D CT views clearly illustrated the axis of the avulsion producing outwardly and downwardly emphasizing the action of the tensor fascia lata (Figures 2 & 3). The patient was successfully treated conservatively.

## Discussion

Apophyseal avulsion injuries of the hip and pelvis have increased in prevalence over the past several decades because more children and adolescents are participating in athletic activities. Apophyseal avulsions account for 10% to 24% of athletic injuries in children. The most commonly implicated activities are soccer, tennis, fencing, track and cross-country but also ballet. Similar injuries also occur in baseball, gymnastics and cheerleading in the US [1,2,3]. In Europe, soccer is the most frequent sport resulting in pelvic avulsions but tennis has emerged as an increasing source of similar injuries. Like soccer, tennis also requires a large range of repeated and sometimes explosive motion in all directions [3].

The patient’s age represents the major factor determining where the disruption occurs in the chain of bone, tendon, and muscle. In a young adult, the failure usually involves the myotendinous junction. In an older adult, the failure tends to target the tendon, which is often weakened by tendinosis. Finally in children and adolescents the weak link is the physis, especially at times of growth acceleration [1].

An apophysis in a child or adolescent is a secondary center of ossification that contributes to the shape or size of a bone but not to its length [1]. An apophysis is connected to its parent bone by a physis. An apophysis is also termed a “traction epiphysis” as it is the site of attachment of muscles or tendons.

The usual mechanism of acute apophyseal avulsion in children or adolescents is an acute indirect injury during which sudden forceful concentric or eccentric muscular contractions pulls the apophysis (at the level of the growth cartilage) rather than the corresponding tendons or muscles that are very strong [1, 3,4,5]. Thus acute apophyseal avulsions are mostly noncontact injuries and typically present with severe and well-localized pain. Other less common mechanisms are direct contact injuries. Finally extreme passive stretching and chronic repetitive microtrauma have also been implicated in the development of apophyseal avulsions.

**Figure 4:**
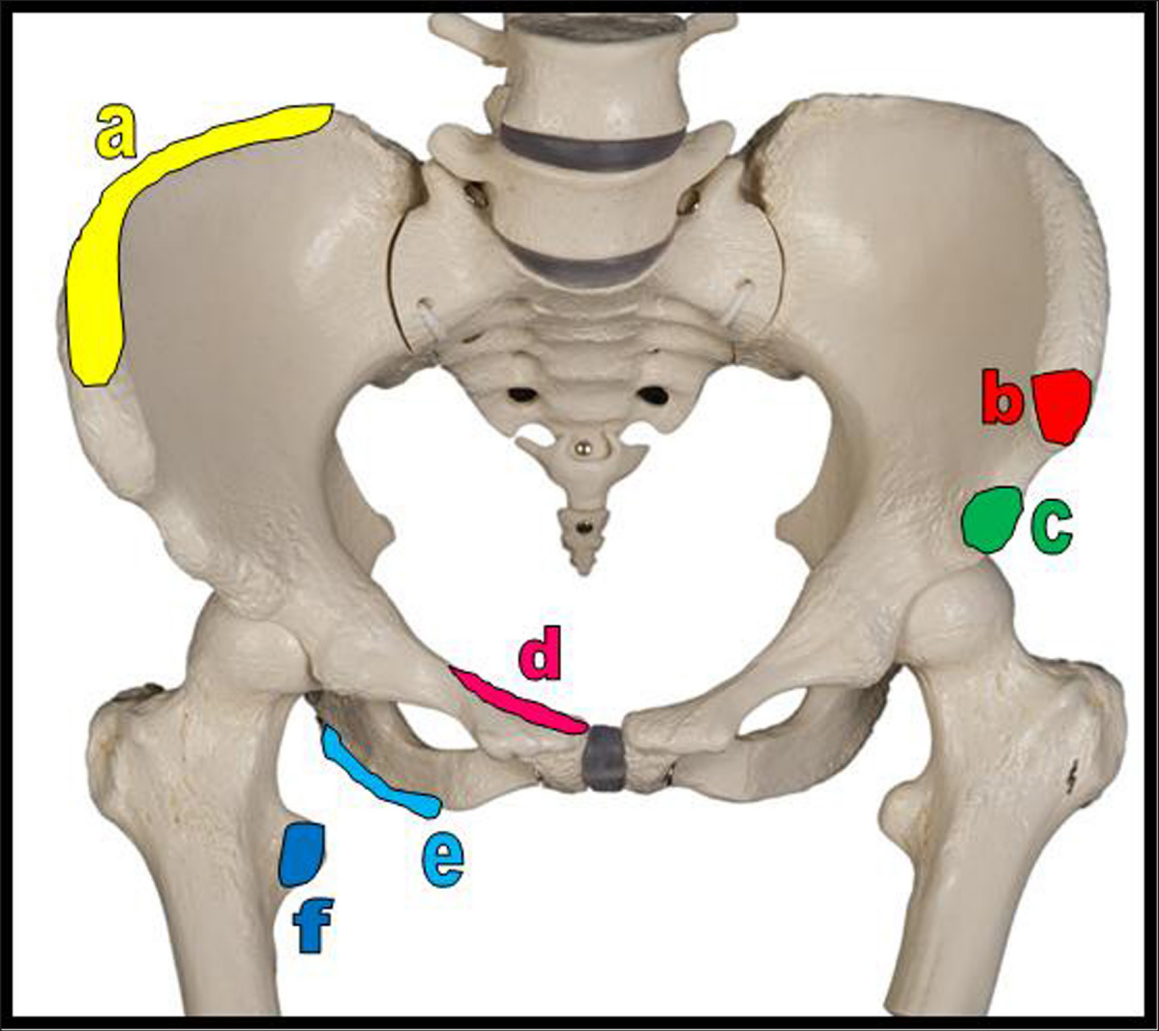
Schema of the most frequent sites of pelvic apophyseal avulsion fractures. a = iliac crest (insertions of the abdominal muscles, the tensor of fascia lata and of the gluteus medius); b = anterior superior iliac spine (insertion of sartorius); c = anterior inferior iliac spine (insertion of rectus femoris); d = superior corner of pubic symphysis (insertion of rectus abdominis); e = ischial tuberosity (insertion of hamstrings muscles = semitendinous, semimembranous and long head of the biceps femoris muscles); f = lesser trochanter (insertion of iliopsoas muscle).

Most apophyseal ruptures or avulsion of the pelvic area (Figure 4) occur at the level of the anterior superior iliac spine (the origin of the sartorius and some fibers of the tensor fascia lata), anterior inferior iliac spine (the origin of the straight head of the rectus femoris), ischiatic tuberosity (the origin of the hamstrings comprising the semitendinous, semimembranous and long head of the biceps femoris muscles), the pubic synthesis (the origin of the adductor brevis, adductor longus, and the gracilis) and the lesser trochanter (insertion of the iliopsoas) [2,3]. Avulsion fractures of the iliac crest apophysis are much rarer representing only 2 % of all pelvic fractures and typically occurs in males (sex ratio 15:1) aged 11–25 years [6].

The iliac crest apophysis remains cartilaginous until adolescence [2]. During this period radiographs may be interpreted as negative. Ossification centers typically appear first along the anterolateral aspect of the iliac crest, at approximately 13–15 years old. Ossification continues in a posteromedial direction toward the posterior iliac spine. Fusion of the ossified apophysis to the iliac bone begins around the age of 15 years but can still produce up to the age of 25 years.

The iliac apophyses serve as the insertion site of the three abdominal lateral muscles comprising the transverse abdominal muscle and the internal and external obliques muscles. A fracture may occur as the result of and increased strain across the growth cartilage of the apophysis caused by sudden lateral flexion contraction and/or twist motion of the lateral abdominal muscles thwarting the antagonist action essentially of the gluteus medius muscles and of the tensor fascia lata [6,7].

The most commonly reported mechanisms in avulsion fractures of the pelvis are kicking (19.7%) and running (40.9%) [8]. In our reported case the avulsion was caused by kicking during indoor soccer.

The biomechanics of kicking in soccer shows that the forward motion of the kicking leg (the right leg in our right-handed player) is initiated by rotating the pelvis around the supporting leg (the left leg of our patient) and by bringing forwards the thigh of the kicking leg. The rotation of the pelvis is greater for the faster kick than for the slower kick. Our patient who was a right-handed indoor soccer explained that he had kicked the ball fast with his dominant right leg at a sharp angle to the left so that he had to rotate his pelvis far to the left while maintaining the upper trunk to the right (Figure 4). This movement is assisted preferentially by the left external oblique. The sudden contraction of the left external oblique probably pulls the apophysis off the left iliac crest. The left internal oblique and the transverses abdominis also insert into the iliac crest and probably contribute to the injury [9]. After avulsion the apophysis was probably displaced laterally and inferiorly, as clearly demonstrated on the 3D views by the antagonist traction of the gluteus medius muscle but merely of the tensor fascia lata [8]. In tennis the sudden contraction of the abdominal muscles caused by a violent rotation of the trunk during power serves and ground strokes may result in the same injury [3].

When avulsion produces most patients have an acute popping sensation immediately associated with acute pain. At physical examination point tenderness over the iliac crest, localized swelling, and a positive Trendelenburg gait attributable to pain and muscle spasm are found [6]. Most patient are neither able to walk nor bear weight [6].

Although he was asymptomatic before the acute injury it is more than likely that chronic mechanical stress of the iliac apophysis had preceded the acute avulsion in our patient. Indeed, hypertrophy and sclerosis of the iliac bone at the base of the physis were clearly visible on axial CT sections illustrating this chronic mechanical overuse.

Plain radiograph is the initial and frequently the only imaging evaluation performed in acute traumatic avulsion. Sonography, MRI, and CT have been used to evaluate acute apophyseal injuries of the pelvis or to complete false negative or ambiguous cases [2,3]. Chronic stress injury – specifically, of the iliac crest - has also been evaluated with scintigraphy and more recently with MRI [2].

Due to the generally young age of the injured patients maximal radiation protection must be guaranteed. Therefore ultrasound or MRI should be strongly preferred to CT. Nevertheless CT remains the best method to detect minimal displacement of avulsed ossified apophysis. It is also the modality of choice to obtain optimal 3D views of bone structures. In the reported case CT with 3D reconstructions was preferred to ultrasound because of the very strong habitus of the patient.

Displacement with pelvic fractures is generally minimal because of the multiple muscle attachments of both the trunk and legs. Therefore, conservative treatment is usually sufficient for full recovery. Nondisplaced or minimally apophyseal avulsions of the pelvis are usually treated with nonsteroidal anti-inflammatory agents, activity modification, and rehabilitation. After resolution of clinical symptoms, which usually takes 4 to 6 weeks, the patient can gradually return to sport activity. Surgery is only considered for recent apophyseal avulsion fractures displaced more than 3 cm or if fragments encroach on nerve or vascular supply [1,6].

## Competing Interests

The author declares that they have no competing interests.

## References

[1] Kjellin I, Stadnick ME, Awh MH (2010). Orthopaedic magnetic resonance imaging challenge: apophyseal avulsions at the pelvis. Sports Health.

[2] Hébert KJ, Laor T, Divine JG (2008). MRI appearance of chronic stress injury of the iliac crest apophysis in adolescent athletes. AJR Am J Roentgenol.

[3] Vandervliet EJ, Vanhoenacker FM, Snoeckx A, Gielen JL, Van Dyck P, Parizel PM (2007). Sports-related acute and chronic avulsion injuries in children and adolescents with special emphasis on tennis. Br J Sports Med.

[4] Kerssemakers SP, Fotiadou AN, de Jonge MC (2009). Sport injuries in the paediatric and adolescent patient: a growing problem. Pediatr Radiol.

[5] Aksoy B, Oztürk K, Ensenyel CZ (1998). Avulsion of the iliac crest apophysis. Int J Sports Med.

[6] Steerman JG, Reeder MT, Udermann BE (2008). Avulsion fracture of the iliac crest apophysis in a collegiate wrestler. Clin J Sport Med.

[7] Lambert MJ, Fligner DJ (1993). Avulsion of the iliac crest apophysis: a rare fracture in adolescent athletes. Ann Emerg Med.

[8] Porr J, Lucaciu C, Birkett S (2011). Avulsion fractures of the pelvis – a qualitative systematic review of the literature. J Can Chiropr Assoc.

[9] Valdés M, Molins J, Acebes O (2000). Avulsion fracture of the iliac crest in a football player. Scand J Med Sci Sports.

